# The Inefficient Cook—From a Matron's Point of View

**Published:** 1912-09-21

**Authors:** 


### INSTITUTIONAL HOUSEKEEPING.

### The Inefficient Cook—From a Matron's Point of View.

How many boards and committees realise that next in importance to the matron comes the cook? The comfort and health of the establishment depends on her. Too often a false economy is practised here, and no one knows this better than the matron herself. A well-trained cook is an invaluable asset, for she is economical in a far wider sense than in the reduction of the tradesmen's books. Having grasped the value of foods, not by nice theories, but by real practical knowledge her economies are far-reaching. She knows that twice-cooked food, however appetisingly and daintily dished, is of diminished value to the worker; she also knows that to those whose mental powers are kept on the stretch for many hours routine diet is an abomination. To know that for endless Wednesdays, say, there is going to be beef-steak pie or roast mutton is more tiring than the most trying "theatre" work.

"Routine cooking" is the standby of the badly-trained cook. She loves fixing up a diet-sheet for the week, which becomes as the law of the Medes and Persians. She is of the squirrel-in-the-cage order; she has no imagination, and, though she does not know it, she is lazy. You will know her by the fact that her gravies have no distinctive flavour. She prospers exceedingly in institutions; the board think highly of her, because they can tell to a half-sovereign what her accounts will come to, and that makes the balance sheet easy if heavy. She is a joy to the store-keeper, for her requisitions never vary, and that saves him a lot of trouble. She is loved by the contractors for the same reason. As far as her limitations go she is quite good, and is, therefore, the more difficult to uproot; but she is the despair of the head of the housekeeping department, for she is the prime cause of discontent and dissatisfaction. Moreover, she is essentially extravagant, for women of this type require a large margin and are immovably obstinate on the subject of waste.

There is another class of cook—the one who takes a small wage and who has to be trained by the housekeeper. She is often obtained through an interested member of the visiting committee, who thinks the place would provide excellent training for the girl. She has most likely been a "general" in a family where there are children, and, having done fairly well, the proposer thinks this would be a good opportunity for her to better herself. It may be said here and now that this usually spells disaster in the kitchen and general discomfort over the whole household, nor is it fair to the housekeeper.

This may seem at first to be put too strongly, but twenty years of managing large institutions, including hospitals, has not yet made the exception known to the writer. We are not afraid to declare that it is possible for the inefficient cook to strain economy to the point of disaster. We recall the case of a young hospital matron in the Colonies who congratulated herself on the marvellously low rate at which her staff was boarded owing to the skill of her cook in making charming little dishes out of odds and ends. It was, indeed, a wonderful figure. But in the same breath she was complaining there had been a perfect epidemic of "throats" amongst her nurses, and she thought she must have collected all the chronics in the nursing profession. Poor staff! Later on the little matron herself had a sudden break-down. It was inevitable; they had all been slowly starved on elegant trifles.

The institution cook has to learn that the woman as well as the man worker requires constant thought and attention where food is concerned, and that the result of this is felt all through the institution, be it hospital, school, or college, for the general health of the worker under these conditions will maintain a high standard, and a healthy worker is a cheerful worker, and a cheerful worker brings brightness and contentment in her train. There is nothing so costly as the inefficient, poorly paid institution cook.

### Housekeeping Queries.

### More Dishes for Convalescents.

Little Fish Cutlets.—Make a fairly stiff white sauce, add any bits of nice fish (cooked), and a little cooked rice if liked, chopped parsley (if allowed), seasoning, and one raw egg; mix all together, slowing on the fire when fairly firm. Put it on a plate to cool, then make into nice cutlet shapes, roll in egg and bread crumbs, and fry a nice brown.

Another of Eggs and Potatoes.—Take as many potatoes as you have eggs—as near the same size as possible. Bake in their skins until done, cut a piece off the side, taking care not to break the skin of the potatoes, scoop out all the inside, put it through a sieve, add butter and milk, make it very soft and season well; put some of the mixture in the cases, lay in a nicely poached egg, trim each one, cover with more of the mixture, dip a knife into water, make the top smooth, sprinkle with bread crumbs, put them back in the oven and serve very hot.

### To Remove Marks from Furniture.

Esther.—Finger marks, which are usually nothing but grease, can be removed from furniture by washing with warm water and a little soap, drying thoroughly, and polishing quickly with furniture cream. Ink may be removed by gently rubbing with a damp cloth and a little salt. Spirit, such as eau-de-Cologne or methylated spirit, removes the polish entirely, but small spots can be treated with a good furniture cream, rubbing it in gently. If the stain is large, re-French polishing will be necessary. Blisters arising from placing hot dishes, etc., on polished surfaces without mats can be removed, if small, by dabbing with linseed oil and allowing to dry. The oil gives the wood back its natural oil and helps to darken the spot, and frequent polishing with cream will make the surface regain its polish. The best cleaning material for white wood is a paste made from equal quantities of soft soap, sand, and whitening dissolved in water over heat (1 lb.—1 qt.) and used like soap on the brush. It cleans without leaving scratches as sand does when used alone, and as the soft soap contains no soda it does not make the wood yellow.

